# The Value and Interpretation of Race and Ethnicity Data in the Era of Global Migration: A Change Is in Order

**DOI:** 10.4269/ajtmh.21-0665

**Published:** 2021-10-11

**Authors:** Serin Edwin Erayil, M. Kumi Smith, Tsige Gebreslasse, Patricia F. Walker, Erin M. Mann, Syreeta Wilkins, William M. Stauffer

**Affiliations:** ^1^ National Resource Center for Refugees, Immigrants, and Migrants, University of Minnesota, Minneapolis, Minnesota;; ^2^ Division of Infectious Diseases and International Medicine, Department of Medicine, University of Minnesota, Minneapolis, Minnesota;; ^3^ Division of Epidemiology and Community Health, School of Public Health, University of Minnesota, Minneapolis, Minnesota;; ^4^ Global Medicine, Department of Medicine, University of Minnesota, Minneapolis, Minnesota;; ^5^ HealthPartners Institute, Bloomington, Minnesota

## Abstract

Human migration and travel are leading to increasingly diverse populations throughout the world. Data collection practices need to adapt to these changes to expand our understanding of health disparities and to optimize the efforts to address health equity, particularly during public health emergencies such as the current COVID-19 pandemic. Race and ethnicity classifications in the United States have failed to evolve since the 1970s despite an increasingly diverse population. Current commonly collected categories are inadequate to accurately describe the economic, educational, and sociopolitical circumstances of different groups. Further, these categories lend little practical information to inform health policy. More predictive and actionable variables should be routinely collected to improve appropriateness and timeliness of health interventions. The immediate adoption of the collection of primary/preferred language and country of birth/origin by public health organizations, health systems, and clinical providers would be a concrete and valuable first step.

Categorization of individuals by race/ethnicity has a fraught history in the United States. Race data has been collected by the United States government since the first census in 1790. However, specific racial categories and justification for collecting these data have fluctuated over time, reflecting the perspectives of the dominant sociopolitical class. Until recently, pervasive disparities across numerous health outcomes were interpreted as evidence of biological or cultural differences across racial/ethnic groups. Today, a growing consensus recognizes that many of these differences instead reflect social determinants of health, as well as structural racism found in nearly every domain of American life.^1^ The COVID-19 pandemic has brought into focus the importance of having actionable health data for public health and health systems to quickly and more equitably recognize gaps, and respond to public health emergencies. This, along with the evolution in understanding of race/ethnicity data demands a critical reassessment of current standards in data collection in healthcare.

Modern travel and migration patterns have facilitated increasingly diverse populations, particularly in common destination countries such as the United States. In the United States, refugees, immigrants, and migrants alone account for approximately 13.7% of all residents, or approximately 45 million people.^1^ The current broad race/ethnicity labels to which these individuals are assigned fail to identify differential risks between these very distinct groups. For example, people of Middle Eastern or North African heritage are currently considered white. However, the disparities they suffer, the racism they encounter, and the health challenges they face, are more akin to those of other people of color.^2^ The current data collection systems also collapse people with distinct cultural, behavioral, language, social, or environmental characteristics critical to health outcomes into single racial categories. For example, the term “Black” combines American descendants of slavery across the United States and the Caribbean, as well as immigrants from African countries. Other factors that decrease the accuracy and usefulness of the current race/ethnicity data collection include a growing number of people identifying as multiracial/cultural, and definitions and conceptualizations of race varying by culture.

Current categories for classifying individuals by race and ethnicity for federal statistics were defined in a 1978 Office of Management and Budget directive, “Race and Ethnic Standards for Federal Statistics and Administrative Reporting.”^3^ This standardization of race/ethnicity data collection has proved crucial in unveiling stark disparities in morbidity, mortality, healthcare access, and utilization across broad racial and ethnic groups.^4^ However, this construct has failed to evolve over the last 40 years and is insufficient in capturing vital information in an increasingly complex United States. Addressing social health inequities demands more granular data that can more precisely identify the groups that are disproportionately impacted by the conditions of concern, are better proxies for underlying drivers of health disparities, and which provide more actionable data to inform public health organizations, health systems, and clinical providers. Data that provide more accurate and meaningful information may result in more effective interventions, as well as more equitable allocation of resources.

The COVID-19 pandemic in the United States serves to highlight some of the shortcomings of the current system. Although it was apparent early in the pandemic that large disparities were linked to race/ethnicity, to date, more than a year into the pandemic, there are limited data distinguishing which specific populations are at greatest risk of being infected by COVID-19 or reporting on characteristics that are essential to developing effective interventions for those populations.^5^ The National Resource Center for Refugees, Immigrants, and Migrants (NRC-RIM) has been tasked with developing resources to aid these populations during a public health emergency. The available data and multiple publications about disparities according to race and ethnicity have provided limited insight into which groups in the United States are at greatest risk of being infected, or how to prioritize the interventions and resource development (e.g., which of the hundreds of primary spoken language materials should be prioritized). The response from NRC-RIM to COVID-19 could have been faster and more efficient if this information would have been available early in the pandemic. To date, attitudes, beliefs, and other barriers about COVID-19 in many patient populations remain largely undescribed even as the ultimate success of contact tracing, mitigation, and prevention efforts, including vaccination, hinge on this information. During the pandemic and more broadly, health interventions will have little of the intended impact when not delivered in the population’s linguistic, cultural, and sociopolitical context.

While comprehensive reform of public health and health systems’ data collection strategies in the United States may be politically and bureaucratically challenging, there appears to be an increased awareness of health disparities and of the shortcomings of the current system. Although challenging, we propose that the routine collection of two variables would greatly improve the usefulness of these data: primary/preferred language and country of birth/origin. Others have advocated for also adding self-defined race/ethnicity data,^6^ which would increase complexity, but we agree would be ideal. HealthPartners care system in the Midwest United States has routinely collected race/ethnicity, preferred language for interacting with the care system, and country of birth/origin for > 15 years, proving both feasibility and acceptance by patients when they are informed of the intended use of these data and assured of data security and confidentiality. In the HealthPartners care system, a multispecialty health system covering more than 1 million patients and including hospitals, clinics, and insurance providers, collection of these two variables has been used to identify and respond to health disparities with a goal of improving population health while reducing costs. Three specific examples of how these data have been used follow:

First, hepatitis B infection disproportionately affects foreign-born individuals. Despite a 2008 CDC recommendation for routine screening, it is estimated that < 30% of affected individuals are currently screened or treated, leading to preventable deaths from liver disease and associated carcinoma.^7^ Country of birth/origin was used to develop a “Global Health Wizard” electronic medical record best practice that alerted clinicians when a patient who should be screened by CDC criteria had not been screened. This program has been used to identify those with “hidden” infections who could then be referred for ongoing care to prevent end-stage liver disease and cancer.^8^

Second, significant disparities by race and country of birth exist for women between ages 50 and 75 who are up to date (UTD) for mammogram screening for breast cancer. In one 2009 sample, UTD results for all white (84.4%) versus all Black (73.9%) women revealed a 10.5% disparity gap. When data were disaggregated by white (84.4%) versus United States–born Black (77.9%) versus foreign-born Black (63%) women, the disparity gap between white and foreign-born Black women was even more profound, at 21.4%. Targeted linguistically and culturally appropriate (e.g., Somali, Liberian) interventions including offering same day mammography and public transportation support to mammography sites have reduced the gap significantly. These data were also used to advocate for and obtain on-site mammography for a clinic serving many African-born women (HealthPartners data, unpublished).

Third, the use of in-person medical interpreters is considered a best practice but frequently not done because of cost. The routine collection of data based on language allowed evaluation of length of stay for inpatient mental health admissions for those who spoke English as a primary language versus those with limited English proficiency (LEP). Length of stay was found to be almost one full day longer for patients with LEP. This information then allowed advocacy toward a standard policy for provision of in-person medically trained interpretation during inpatient mental healthcare, which was associated with reduction in that disparity, and a decrease in cost of care (HealthPartners Interpretive Services, unpublished data).

When used for public health, additional collected data can be analyzed with other factors associated with risk such as health insurance, zip code/zip code tabulated areas, or other frequently collected proxies for social determinants of health. The ability to stratify granular demographic data allows for powerful insights and more targeted and effective interventions. Ideally, these data would be collected consistently across the continuum of care, from public health surveillance to health systems, and at the clinical interface. Although these changes would require agreement, standardization, and significant investment to update data collection tools and data systems, the downstream public health and patient care benefits could be significant.

Although collection of primary/preferred language for interacting with a care system and country of birth/origin is a straightforward concept it does present challenges. For example, it would require buy in from public health agencies, health care systems, and providers. Other issues such as standardizing data, determining the point/s of care at which such data are collected, staff training on data collection and messaging for patients, and methods to manage the large number of languages and countries of origin all present challenges. Some of these issues could be addressed through community engagement. Communities should be involved in providing input such as defining terms and assisting in suggestions around implementation. This would increase trust and support and would likely improve the chances of success. There is also the threat of malevolent use or misuse of data both from government and non-government actors in the form of anti-immigrant policies and violent acts which, unfortunately, have been increasing across the United States. Individuals should be able to opt-out and data must be protected by the strict use, and strengthening, of data standards. In addition, the healthcare and scientific community must stand firm against misuse of data, racism, and xenophobia. There will be additional limitations to data collection and the model we propose will still not always accurately identify certain populations/groups (e.g., if a patient is Black, Portuguese-speaking, and Brazilian, reporting only country of origin and language would create an incomplete profile). However, other changes to data collection could continually improve the system such as the suggested collection of self-identified ethnicity.^6^

While countries differ in their philosophy and approach to collecting public health and healthcare system data, we believe collecting more granular data can be used to improve appropriateness of health interventions and patient care, and ultimately, further address the undeniable health impacts of the historical legacy of racial discrimination. As practitioners of Global Medicine in the United States, we have an increasing call to engage in reevaluation of practices that might have had their origin in colonial medicine.^9^^–^^11^ Refining race/ethnicity data collection practices could improve our ability to respond to public health crises, more equitably and cost effectively direct resources, and substantially impact health disparities. A change is long overdue.
